# Science Diplomacy as an Umbrella Term for Science Advisory in Public and Foreign Relations in Small Developing Countries: The Case of Panama

**DOI:** 10.3389/frma.2021.655335

**Published:** 2021-04-12

**Authors:** Rolando A. Gittens, Sandra Lopez-Verges, Thais Collado, Jennifer Pimentel, Anabella Vazquez, Marta Pulido-Salgado, Ivonne Torres-Atencio

**Affiliations:** ^1^ Centro de Biodiversidad y Descubrimiento de Drogas, Instituto de Investigaciones Científicas y Servicios de Alta Tecnología (INDICASAT AIP), Panama, Republic of Panama; ^2^ School of Medicine, Universidad de Panama, Panama, Republic of Panama; ^3^ Gorgas Memorial Institute of Health Studies, Panama, Republic of Panama; ^4^ Ministry of Foreign Affairs, Panama, Republic of Panama; ^5^ Secretaria Nacional de Ciencia, Tecnología e Innovación, SENACYT, Panama, Republic of Panama; ^6^ Investigación y Ciencia (Spanish Edition of Scientific American, Springer Nature), Barcelona, Spain; ^7^ Pharmacology Department, School of Medicine, Universidad de Panama, Panama, Republic of Panama

**Keywords:** science advice, legal framework, science diplomacy, sustainable development, science ecosystem, evidence-based decision making, public policy, foreign policy

## Introduction

The history of Panama has been influenced by science and technology since its inception. The geological event that formed the isthmus that united North and South America, more than 3 million years ago, gave it its strategic location and one of the greatest biodiversity riches in the world (O’Dea et al., 2016). The construction of the Panama Canal was one of the most ambitious engineering works of its time, but the poor management of workers’ health conditions played a key role in the failure of the original project, led by the French (Marshall, 1913). When the United States took over the work, right after Panama’s independence from Colombia in 1903, Dr. William Gorgas implemented measures similar to those taken in Cuba following Dr. Carlos Finley’s research to clear yellow fever mosquitoes (Mason, 1916; Chaves-Carballo, 2005). This allowed the successful completion of a great work of engineering, becoming one of the first examples of Science Diplomacy (SD) for global health.

Despite the complex diplomatic relationship between the US and Panama, both countries agreed to establish in Panama some of the best institutes in Tropical Ecology and Medicine, like the Smithsonian Tropical Research Institute (STRI) and the Gorgas Memorial Institute (GMI). Implemented by the US but officially administered by the Panamanian Government since 1990, the latter has become an international reference institute for tropical and public health research (Wright, 1970; Adames, 2003). STRI continues to be an American institute that studies all aspects of the abundant and untapped biodiversity in the country (STRI, 2018).

Many of the most relevant historical scientific and technological events in Panama are intertwined with political decisions and have occurred generally under the leadership of another country, or were undertaken by an interested group of scientists and technicians and not as a national strategy. It was not until the end of the 20th century, that Panama began to systematically develop and strengthen its scientific ecosystem with the creation of the National Secretariat of Science, Technology and Innovation (SENACYT) (Romero and Quental, 2013). Currently, it is the main public institution funding research and the first to promote the importance of training young scientists in communication, relational and leadership skills in SD (Gittens and Lopez-Verges, 2018). SENACYT also encourages the interaction between the scientific community and the Ministry of Foreign affairs, as well as researchers’ involvement in different national strategies and projects.

## Panama's Scientific Development in Its Historical Context as a Bridge for Science Diplomacy

The concept of Panama as a nation is closely linked to diplomatic relations and, although not called as such at the time, SD. First, to achieve the construction of major engineering works, such as the Transisthmian Railway or the interoceanic route, which helped with the desired political separation from Colombia. And once independent, to gain sovereignty in the governance of its territory without intervention from the United States in the Canal Zone, after decades of social unrest due to their presence that led to the loss of Panamanian lives and severed diplomatic relations. This, undoubtedly, has marked how Panama has built its scientific and technological development and international collaborations.

The strategic geographical position of the isthmus has allowed communication and people exchange from all over the world. This, together with Panama’s great biodiversity, has laid the foundations of the scientific institutions that the country has nowadays. The presence of STRI in Panama exemplifies this. During the construction of the Canal, American engineers built a dam on the Chagres River, near the Caribbean coast, to create Gatun Lake and facilitate the passing of ships. The largest human-made lake in the world, at the time, allowed the study of the rainforest biodiversity in newly created islands, like Barro Colorado, and attracted scientists from all around the world (STRI, 2018). STRI is a unique example of a cutting-edge scientific research institute based in the tropics. The collaboration between Panama and the United States, which has lasted more than a century, has been key. STRI is an institution that contributes to global knowledge, something of great relevance in these moments of crisis due to climate change (STRI, 2018).

GMI constitutes another relevant historical case. In 1921, Panama’s President Belisario Porras created it, as a tribute to Dr. William C. Gorgas. In 1928, the institute was inaugurated in Panama City and was administered by the US until 1990. GMI is devoted to research on tropical medicine. Its main focus includes parasitic diseases such as malaria, toxoplasmosis, leishmaniasis and Chagas disease, as well as diseases produced by arboviruses, retroviruses, papillomaviruses and respiratory viruses, among others (Wright, 1970; Sanchez, 1974). Its scientific activity has produced over 1139 publications (ICGES, 2021).

Panama, as a Republic, has had a close, and at certain points complex, diplomatic collaboration with the United States and neighboring countries. This has fostered the exchange of knowledge, technology and trained human resources, which have derived in the basis of our SD, established as a strategy a few years ago.

## Strategies to Establish the Pillars of Science Diplomacy in Panama

In 2018, on the occasion of the Day of the Panamanian Diplomat, the Government, through the Ministry of Foreign Affairs (MOFA), launched the "National Strategy for Science, Technology and Innovation (STI) Diplomacy", as an instrument for the 21st Century diplomacy (SENACYT, 2018; SENACYT and MIRE, 2019). This effort, led by SENACYT and MOFA, allowed Panama to become the first Latin American country with a national strategy on SD.

According to the Vice-President and Minister of Foreign Affairs at the time, Isabel de Saint Malo de Alvarado, the combination of science and diplomacy is accountable for relevant milestones. For instance, the agreement between the United States and the Organization for the Prohibition of Chemical Weapons to destroy the old chemical munitions, left in San Jose Island by the United States military (Pugliese, 2002; AFP, 2019) or the approval of new navigation routes in Panamanian waters to minimize collisions between ships and migrant humpback whales by the International Maritime Organization (Guzman et al., 2013; Cogley, 2014).

The current Government Administration of President Laurentino Cortizo, in charge since July 2019, has created for the first time a Science Cabinet that includes participation from all relevant ministries and is under the coordination of SENACYT. It has established a Technical-Legal Committee, comprising representatives of the different public and research institutions, that is currently working on a draft law to update the legal framework for SENACYT and the National STI System. One of the chapters relates to the concept of SD as an umbrella term for science advisory.

MOFA, together with SENACYT, is working on this action plan aimed to bridge the gap between key stakeholders to promote strategic investment in major projects that will benefit the Panamanian population. Namely:

- The establishment of a pharmaceutical hub in Panama.
- Initiatives for the conservation, restoration and use of invaluable biodiversity, threatened by climate change, to protect Panamanian communities that face rising sea levels and more extreme weather events.
- Biotechnological startups able to implement a new bioeconomy and the need to guarantee Panama's water and food security through a vigorous and competitive agro-industrial sector.
- Biomedical innovations to improve citizens’ quality of life against the rising incidence of infectious and chronic diseases in the population, aggravated by aging.
- Research platforms for the generation of knowledge as a tool to improve education quality in Panama.

## Discussion

The concept of SD, almost unknown five years ago by both scientists and diplomats in Panama, has gone through a broad inter-institutional and inter-sectoral discussion during the establishment of the national strategy for SD. On the one hand, this exercise brought together the scientific and technological ecosystem main actors to define a unified vision of the priorities and strengths in STI for the country, so that Panamanian diplomats could better promote national interests. On the other hand, it made the scientific community visible to diplomats and foreign policymakers, but also to most governmental institutions and civil society that could benefit from the local generation of knowledge. These interactions between scientists, public institutions and civil society have helped to create strong and lasting relationships that are leading to science advisory opportunities for evidence-based public policy (CCIAP, 2019; Redaccion, 2020).

Partially because of its relatively young scientific community, the concept of SD was discussed and implemented before the country had any real science advisory legal framework to use scientific knowledge to inform decision-makers. For this reason, the term “Science Diplomacy” has become a synonym for “Science Advice”. Therefore, the Technical-Legal Committee of the Science Cabinet is considering introducing a chapter in the new law for the National STI System that will combine both terms. A small country such as Panama has traditionally imported technology and even science expertise in the form of foreign scientific consultants for public policymaking or business decision-making. The goal is that this new legal framework will provide diplomats and decision-makers with more access to local scientific expertise.

By using the soft power of science in policy advice, the growing community of science diplomats can boost Panama’s development and take part in different issues at national, regional or global scale. The implementation of SD in Panama offers a ray of light to banish the infamous expression "no one is a prophet in their own land" suffered by many countries in the region. There is a long way to go, but if Panama manages to follow the path set up by its national strategies, it will demonstrate that small middle-income countries can be key players in designing a new multilateral partnership for sustainable and equitable development.
